# Targeting Lactate Metabolism by Inhibiting MCT1 or MCT4 Impairs Leukemic Cell Proliferation, Induces Two Different Related Death-Pathways and Increases Chemotherapeutic Sensitivity of Acute Myeloid Leukemia Cells

**DOI:** 10.3389/fonc.2020.621458

**Published:** 2021-02-05

**Authors:** Ernestina Saulle, Isabella Spinello, Maria Teresa Quaranta, Luca Pasquini, Elvira Pelosi, Egidio Iorio, Germana Castelli, Mattea Chirico, Maria Elena Pisanu, Tiziana Ottone, Maria Teresa Voso, Ugo Testa, Catherine Labbaye

**Affiliations:** ^1^ National Center for Drug Research and Evaluation, Istituto Superiore di Sanità, Rome, Italy; ^2^ Core Facilities, Istituto Superiore di Sanità, Rome, Italy; ^3^ Department of Oncology and Molecular Medicine, Istituto Superiore di Sanità, Rome, Italy; ^4^ Department of Biomedicine and Prevention, University of Rome “Tor Vergata”, Rome, Italy; ^5^ Santa Lucia Foundation, I.R.C.C.S., Neuro-Oncohematology, Rome, Italy

**Keywords:** acute myeloid leukemia, lactate metabolism, MCT1, MCT4, AR-C155858, syrosingopine, autophagy

## Abstract

Metabolism in acute myeloid leukemia (AML) cells is dependent primarily on oxidative phosphorylation. However, in order to sustain their high proliferation rate and metabolic demand, leukemic blasts use a number of metabolic strategies, including glycolytic metabolism. Understanding whether monocarboxylate transporters MCT1 and MCT4, which remove the excess of lactate produced by cancer cells, represent new hematological targets, and whether their respective inhibitors, AR-C155858 and syrosingopine, can be useful in leukemia therapy, may reveal a novel treatment strategy for patients with AML. We analyzed MCT1 and MCT4 expression and function in hematopoietic progenitor cells from healthy cord blood, in several leukemic cell lines and in primary leukemic blasts from patients with AML, and investigated the effects of AR-C155858 and syrosingopine, used alone or in combination with arabinosylcytosine, on leukemic cell proliferation. We found an inverse correlation between MCT1 and MCT4 expression levels in leukemic cells, and showed that MCT4 overexpression is associated with poor prognosis in AML patients. We also found that AR-C155858 and syrosingopine inhibit leukemic cell proliferation by activating two different cell-death related pathways, i.e., necrosis for AR-C155858 treatment and autophagy for syrosingopine, and showed that AR-C155858 and syrosingopine exert an anti-proliferative effect, additive to chemotherapy, by enhancing leukemic cells sensitivity to chemotherapeutic agents. Altogether, our study shows that inhibition of MCT1 or MCT4 impairs leukemic cell proliferation, suggesting that targeting lactate metabolism may be a new therapeutic strategy for AML, and points to MCT4 as a potential therapeutic target in AML patients and to syrosingopine as a new anti-proliferative drug and inducer of autophagy to be used in combination with conventional chemotherapeutic agents in AML treatment.

## Introduction 

Cancerous tissue and cells run high rate of glycolysis and rely on pyruvate reduction to lactate for energy production to support their proliferation and anabolic growth under oxidative stress and hypoxia, or under aerobic conditions, a phenomenon referred to as “the Warburg effect” (1–3). Thus, altered energy metabolism is regarded as a hallmark of cancer and targeting glycolysis is currently under investigation for cancer therapy (1–5). Highly glycolytic cancer cells prevent intracellular acidification due to increased metabolic activity by excreting the excess of glycolytic end-products lactate and H^+^ *via* the monocarboxylate transporters (MCTs) pertaining to the larger Solute Carrier 16 (SLC16) family (1–6). MCTs play an important role in cancer metabolism and in particular MCT1 and MCT4 that are overexpressed in cancer cells and represent promising targets for anticancer therapies (7–9). MCT1 mediates the bidirectional high-affinity transport of monocarboxylates, including lactate, pyruvate, acetate, and D,L-b-hydroxybutyrate while MCT4 mediates the export of lactate in highly glycolytic cells (6, 10). Both MCT1 and MCT4 require the association with the multifunctional transmembrane glycoprotein CD147, also called basigin or EMMPRIN (Extracellular Matrix MetalloPRoteinase Inducer), for their correct functionality and translocation to the plasma membrane where they act (11–14). MCT1 and MCT4 are also involved in the proper expression of CD147 in the plasma membrane (11–13). CD147 co-overexpression with MCT1 and MCT4 correlates with biological functions that promote tumor progression and confers resistance to chemotherapeutic drugs (12, 13). Like its chaperone CD147, MCT4 is hypoxia-dependent and CD147-MCT4 co-overexpression is regarded as unfavorable prognostic factor in cancers associated with hypoxia, a common feature of solid tumors but also a major component of the bone marrow microenvironment crucial in leukemia progression (14–18). Recently, we showed that CD147 is overexpressed in acute myeloid leukemia (AML) and demonstrated the prognostic value of its expression level in AML blasts, as previously shown in multiple myeloma (MM) cells (19–22). However, while the CD147-MCT1 transmembrane complex is required for MM cells proliferation, the understanding of the role of CD147-MCT1 and CD147-MCT4 complexes is still incomplete and MCT1/MCT4 activities poorly defined in AML (19–21). Indeed, the metabolism of AML blasts differs from most other cancers in that AML is primarily dependent on mitochondrial oxidative phosphorylation (OXPHOS) for growth and survival (17, 18). However, AML is a heterogeneous clonal disorder whose clones have the common feature of all acute leukemia, i.e., a rapid proliferation and high metabolic demand that can also depend on glycolysis in aerobic conditions (23–25). Thus, by using several metabolic strategies and different sources of nutrients for energy supply, AML cells gain metabolic plasticity adapting to oncogenic and environmental changes that result in continuous growth and drug resistance (25). Recently, we also demonstrated that CD147, whose overexpression promotes leukemic cells proliferation, is a potential therapeutic target in AML patients and its inhibitor AC-73 is an anti-proliferative drug that induces autophagy in leukemic cells (22, 26). Here, we analyzed MCT1 and MCT4 expression and function in normal and leukemic cells. We explored the use of their inhibitors, AR-C155858 (AR-C), a specific inhibitor of MCT1 and MCT2, and syrosingopine (SYRO), previously described as a dual MCT1 and MCT4 inhibitor but with a major affinity for MCT4, to reduce glycolysis and evaluate their effects on leukemic cell proliferation *in vitro*, in combination or not, with the more conventional chemotherapeutic agent used in myeloid leukemia, arabinosylcytosine (Ara-C) (8, 27, 28).

Altogether, our data show that MCT1 and MCT4 play a key role in leukemic cell proliferation and suggest that lactate metabolism is a potential target for AML therapy, particularly through MCT4 inhibition by SYRO, an inducer of autophagy with a major affinity for MCT4 whose overexpression is correlated with poor prognosis in AML, to be used in combination with conventional chemotherapeutic agents as a novel treatment strategy in AML.

## Materials and Methods

### Human Cord Blood CD34^+^ HPCs Purification and Culture for *In Vitro* Unilineage Granulocytic (G) and Monocytic (Mo) Differentiation

Human cord blood (CB) were obtained from healthy donors after informed consent and approval by local ethical committees of Istituto Superiore di Sanità, Rome (file number # 171639).

CB CD34^+^ HPCs were purified and cultured in BIT 9500 serum-free medium (Stemcell Technologies Inc. Vancouver, BC, Canada) supplemented with; (i) IL-3 (1 U/ml), GM-CSF (0.1 ng/ml), and G-CSF (500 U/ml) (PeproTech Inc., NJ, USA) for G cultures; (ii) human low density lipoprotein (40 μg/ml), FLT3 ligand (100 ng/ml), IL6 (10 ng/ml), and M-CSF (50 ng/ml) for Mo cultures, as previously described (29, 30). For morphologic analysis, HPCs were smeared on glass slides by cytospin centrifugation, stained with May-Grünwald-Giemsa and analyzed at 400X magnification under a microscope (Eclipse 1000, Nikon, Tokyo, Japan) equipped with a digital camera.

### CD16+/- Monocytes Subset Purification

In vitro Mo-differentiating HPCs, harvested at day 13 of Mo cultures (30), were stained with PE-labeled anti-CD16 antibody and sorted according to fluorescence intensity into CD16 positive and CD16 negative fractions by using a FACS Vantage (Becton Dickinson).

### Human AML Cell Lines Culture and Differentiation

All leukemic cell lines were cultured in RPMI-1640 medium supplemented with 10% FCS (Gibco, Carlsbad, CA, USA), 100 μg/ml streptomycin, 100 U/ml penicillin, in a humidified atmosphere containing 5% CO_2_ at 37°C. AML cell lines used were: U937 as a model of AML-M5, NB4, and HL-60, which is capable of differentiating through the FAB M2/M3 and M4/M5 immunophenotypes as models of AML-M3 and AML-M4, respectively; NB4-R4 as AML-M3 resistant to *all-trans* retinoic acid (ATRA) treatment; MV4-11 as AML-M5 mutated for *FLT3*-ITD. Monocytic-induced differentiation of U937 cells was performed by using 50 ng/ml 1α250H-Vitamin D3 (vit.D3) (Roche, Basel, Switzerland). Granulocytic-induced differentiation of U937 and NB4 cells was performed by using respectively, 10 nM TPA (Sigma) for U937 and 1 µM All-Trans Retinoic Acid (ATRA) for NB4 cells (Sigma), for the indicated time points.

### Human Primary AML Blasts Culture

By using Ficoll-Hypaque density gradient, leukemic blasts were isolated from bone marrow obtained from patients with newly diagnosed AML, after informed consent, according to the Declaration of Helsinki and approval by local ethical committees of Tor Vergata University (file number RS 34.20 del 26/02/2020 Fondazione PTV Policlinico Tor Vergata, Rome). Leukemic blasts were maintained in culture in Iscove’s medium supplemented with 10% FCS, GM-CSF (10 ng/ml), SCF (50 ng/ml), IL-3 (10 ng/ml), as described (30).

### Cell Growth, Cell Cycle Profile, Viability, and Apoptosis Analysis

Cell viability, proliferation, apoptosis, and cell cycle profiles were investigated in HPCs and leukemic cells, treated and control. Cell growth was analyzed by cell counting using trypan blue the rate of proliferation and the percentage of viable cells. Cell Titer-Glo Luminescent Assay (Cell Titer Glo Cell viability assay, G7571; Promega, Madison, WI) was used as a cell viability assay to determine cell growth and the number of viable leukemic cells in culture based on quantitation of the ATP present, an indicator of metabolically active cells, according manufacturer’s procedures. Apoptosis was analyzed by using Annexin V-FITC and Propidium Iodide apoptosis kit to detect both early and late apoptosis, according manufacturer’s instruction (BD Pharmingen, San Diego, CA). Cell cycle analysis were evaluated by using assays according manufacturer’s procedures (7-aminoactinomycin D and Cycletest Plus DNA detection kits, are from BD Pharmingen).

### Clonogenetic Capacity of G and Mo HPCs

Clonogenic assays were performed with CD34^+^ HPCs treated with 1 µM AR-C or SYRO, as compared to untreated (C) CD34^+^ HPCs, in methylcellulose cultures under conditions favoring G and Mo proliferation and differentiation, as previously described (22, 29, 30).

### Flow Cytometry Analysis

Expression of CD14 and CD15 cell surface antigens, stained with PE-conjugated anti-mouse CD14 and CD15 antibodies (BD Pharmingen, San Diego, CA), was analyzed by flow cytometry using a FACScan Flow cytometer (Becton Dickinson, Bedford, MA) to control respectively monocytic and granulocytic differentiation, as previously described (22, 29, 30). FACSCAN flow cytometer with Cell Quest software (BD) was used for acquisition and analysis. The results were expressed in terms of the percentage of positive cells and of the mean fluorescence intensity (MFI).

### Quantitative Real-Time RT-PCR

Total RNAs were extracted using TRIzol reagent and reverse transcribed, as described (30). Quantitative real-time RT-PCR analysis (qRT-PCR) was performed and normalized with the internal control β-actin (ACTB). We used commercial ready-to-use primers/probe mixes for MCT1 (SLC16A1 assay ID Hs01560299_m1), MCT4 (SLC16A3 assay ID Hs00358829_m1), and ACTB (assay ID Hs 9999903_m1) (Assays on Demand Products, Applied Biosystems), according to the manufacturer’s procedures, TaqMan technology and the ABI PRISM 7700 DNA Sequence Detection System (Applied Biosystems, Foster City, CA, USA).

### Western Blot Analysis

Aliquots of 30 µg of total protein extract were prepared and resolved on 10% mini-Protean TGX precast gels (Bio-Rad, CA, USA) for standard denaturing electrophoresis, according manufacturer’s instructions. Precast gels were then transferred to nitrocellulose filters by using Transblot-Turbo transfer system, according manufacturer’s procedures (Bio-Rad, Hercules, CA, USA). Membranes were treated and incubated with specific antibody, as previously described (22). Antibodies used were: MCT1 polyclonal antibody (PA5-78169, Invitrogen/Thermo Fisher Scientific, MA, USA) and MCT4 monoclonal antibody (MCT4 (F10) sc-376101, Santa Cruz,USA); for LC3-I and LC3-II proteins detection, LC3B polyclonal antibody (NB600-1384, Novus Biologicals, Novus, I); for HMGB1, HMGB1(D3E5) monoclonal antibody (#6893, Cell Signaling Technology, MA, USA); monoclonal antibody anti-actin (Sigma-Aldrich, Milan, I) was used as an internal control of the loaded amounts of total proteins.

### Cyto-ID Autophagy Detection

We used an autophagy detection kit (CYTO-ID Autophagy detection kit, ENZ-51031-K200, Enzo Life Sciences, NY, USA) for monitoring autophagy by flow cytometry analysis in live cells treated with (i) AR-C or (ii) SYRO, as compared to control cells. First, control and treated leukemic cells were seeded in triplicate (15.0 x 10^4^ cells/ml in wells of a 6 wells plate)*;* then cells were harvested and prepared according to manufacturer’s instructions prior to analysis by flow cytometry FACScan.

### Metabolomics Analysis by NMR Spectroscopy

Intracellular/extracellular metabolome preparation and Metabolomics analysis by NMR spectroscopy. Deuterated reagents [methanol (CD3OD), chloroform (CDCl3)] and deuterium oxide (D2O) (Cambridge Isotope Laboratories, Inc.) and 3-(trimethylsilyl) propionic-2,2,3,3-d4 acid sodium salt (TSP) (Merck & Co, Montreal, Canada) were used for extraction of aqueous and organic metabolites from leukemic cells treated, or not, with AR-C and SYRO. All collected pellet and respective leukemic cell culture medium were stored at -80°C until metabolomic analysis by NMR spectroscopy was performed. Briefly, for samples preparation related to the intracellular metabolome, leukemic cells, treated or not with AR-C and SYRO, were resuspended in ice-cold extraction solvents [methanol/chloroform/water (1:1:1)] and vigorously vortexed. At least 24 h after, polar and lipid phases were separated by centrifugation at 20,000 x g at 4°C for 30 min. The polar methanol/water phase containing water soluble cellular metabolites was lyophilized by using a rotary evaporator (Savant RTV 4104 freeze dryer), while the organic phase (lipid phase) was collected in tube and chloroform was evaporated under nitrogen gas flow. Both phases of extracts obtained for each leukemic cell lines, treated and not, were stored at -20°C. For the preparation related to the extracellular metabolome, leukemic cells culture media extraction were performed by adding ice-cold extraction solvent (10 volumes of ethanolic solution (EtOH:H_2_O, 77:23, v/v) to each tubes and stored at -20°C for at least 24 h. Afterwards the samples were centrifuged at 14,000× *g* for 30 min and the supernatant obtained was then freeze-dried in a Savant RTV 4104 freeze dryer. The aqueous fractions from cells and residues obtained from media were reconstituted in 700 μl D2O using TSP (0.1mM) as NMR internal standards whereas lipid fractions from cells were resuspended in a CD3OD/CDCl3 solution (2:1 v/v) with 0.05% of tetramethylsilane (TMS), as internal control (31). Then, High-resolution 1H-NMR analysis was performed at 25°C at 400 MHz (9.4 T Bruker AVANCE spectrometer; Karlsruhe, Germany, Europe) on aqueous and organic cell extracts using acquisition pulses, water pre-saturation, data processing, and peak area deconvolution, as previously described (31). The absolute quantification of metabolites, determined by comparing the integral of each metabolite to the integral of reference standard TSP and corrected by respective proton numbers for metabolite and TSP, was expressed as nmoles/10^6^ cells and plotted as fold increase relative to the control.

### ATP and L-Lactate Level Assessment

ATP and L-Lactate levels detection. ATP levels were measured by lysing AR-C-, SYRO-, AC-73- or Ara-C- treated leukemic cells with CellTiter-Glo reagent (Promega) and measuring released luminescence with a luminometer. Experiments were set up in 96-well format (4,000 cells/50 µl medium) and at the desired dose and time-point, ATP content was determined with 50 µl CellTiter-Glo Reagent. Lactate levels were measured enzymatically in 96-well plates according to manufacturer’s specifications (Lactate-Glo Assay, Promega, J5021). Fifteen thousand cells/well or 30,000 cells/well were seeded for U937 and MV4-11 cells. After compound incubation, cells were washed with PBS and lysed with 22.5 µl HCl (0.2N). Cell lysates were neutralized with 7.5 µl 1M Tris-base and incubated with 30 µl detection reagent. Luminescence was recorded after 1 h and intracellular lactate concentrations determined from a standard curve. Extracellular lactate production was measured in the medium with background subtraction from fresh medium. Lactate levels were normalized to protein content (Pierce BCA, 23225).

### Drugs Treatment

AR-C155858 (AR-C) and syrosingopine (SYRO) (Sigma-Aldrich, I) were dissolved in DMSO and diluted in DMEM, with a final DMSO concentration of no more than 0.2% for all *in vitro* studies. In leukemic cell lines, dose-response and time-course analysis were performed by using AR-C or SYRO at 1.0; 2.5; 5.0 and 10 µM from 1 to 4 days of treatment; results were compared with 0.2% DMSO-treated leukemic cells, indicated as control leukemic cells. AR-C and SYRO were added in cultures every 2 days to maintain their activity. Some experiments were performed with AC-73 used at 2.5 or 10 µM, as described (22). AR-C and SYRO were used at 1 µM, in combination with different concentration of Ara-C (from 0.01 to 0.1 µM for U937 cells; from 0.1 to 1.0 µM for MV4-11 cells).

### TCGA Data Analysis

Data set of The Cancer Genome Atlas research network were processed and obtained directly from the public access data portal (http://tcga-data.nci.nih.gov/).

### Statistical Analysis

Student t-test was applied to assess statistical significance between different experimental groups. Data were analyzed by using GraphPad Prism software. For univariate survival analysis, Kaplan–Meier plots with a log-rank test were presented using the overall survival data of AML patients from the TGCA.

## Results

### MCT1 Expression Decreases While MCT4 Increases During *In Vitro* G and Mo Differentiation of Normal HPCs and Leukemic Cells

**Figure 1:**
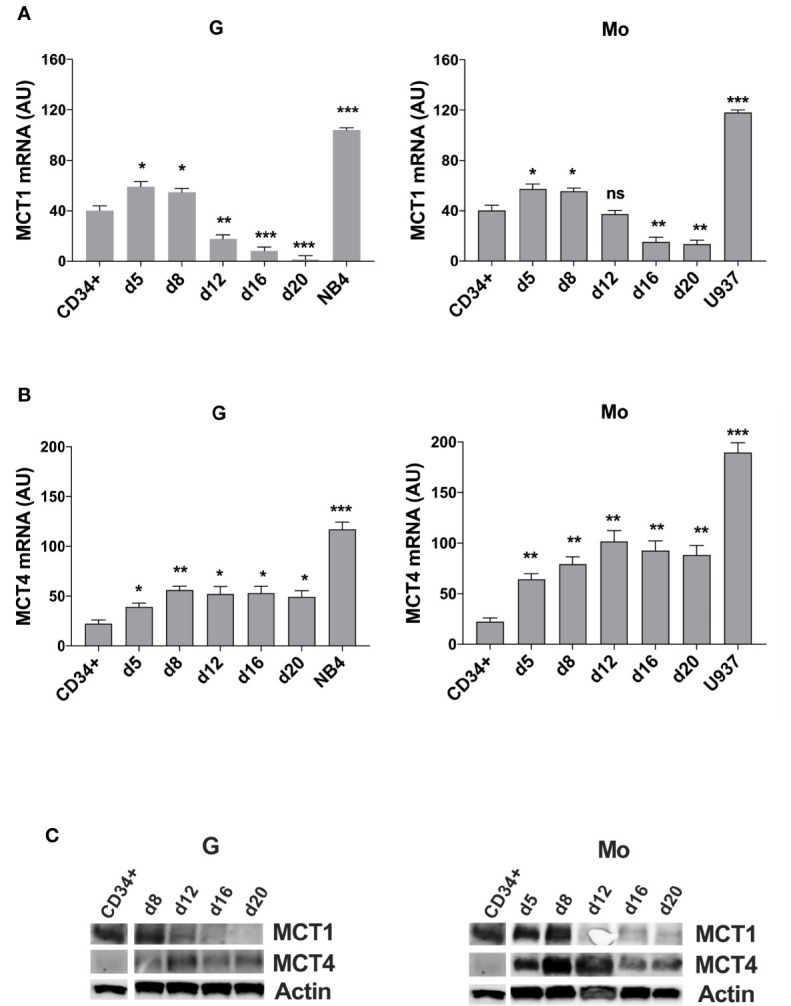
MCT1 and MCT4 mRNA are inversely expressed during Mo and G differentiation of CD34^+^ HPCs. **(A, B)** qRT-PCR analysis of MCT1 and MCT4 mRNA expression during selective G and Mo proliferation and differentiation of CD34^+^ HPCs, as compared to NB4 and U937 leukemic cells; AU is for arbitrary units; the results of three independent experiments (mean ± SEM values) are shown; significance is *p <0.05; **p <0.01; ***p <0.001; ns is for no significant. **(C)** Western blot analysis of MCT1 and MCT4 protein expression during G and Mo proliferation and differentiation of CD34^+^ HPCs. Actin is shown as an internal control; one representative western blot experiment out of three is shown.

First, we analyzed MCT1 and MCT4 expression during *in vitro* G and Mo proliferation and differentiation of normal CD34^+^ HPCs. Expressed in CD34^+^ HPCs, MCT1 increases during the first steps of Mo and G proliferation and differentiation of HPCs (from CD34^+^ day 0 to day 8) and decreases during later steps of differentiation and maturation of these cells (day 12 to day 20) (**Figures 1A, C** **)**, as previously observed for CD147 (22). MCT4 expression, almost undetectable in CD34^+^ HPCs, increases during G and particularly Mo proliferation and differentiation (**Figures 1B, C** **)**.

We also purified CD16^+^ and CD16^-^ cells from day 13 Mo-differentiating HPCs and found an inverse expression between MCT1/4 mRNAs, MCT4 expression level being higher than MCT1 in more mature CD16^+^ monocytes as compared to immature CD16^-^ monocytes (**Supplementary Figure 1**), in line with a previous study (32).

**Figure 2:**
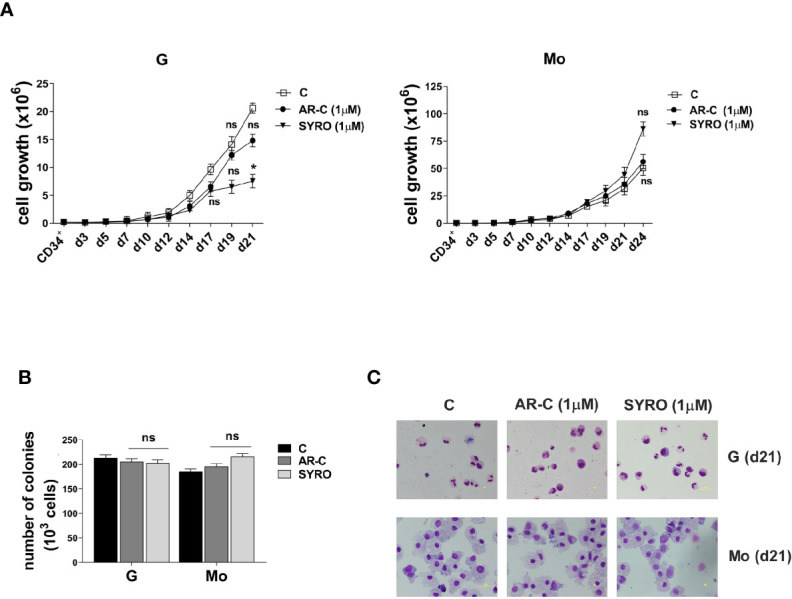
AR-C and SYRO treatment has no major significant effect on cell growth, differentiation and cell cycle progression of G and Mo differentiating HPCs. **(A)** Cell growth analysis during G and Mo differentiation of HPCs, in presence of AR-C or SYRO, used at 1 µM and added every 2 days in cultures, as compared to control (c) cells. **(B)** Clonogenic assays performed under G and Mo culture conditions with AR-C- or SYRO- treated HPCs, as compared to untreated G- and Mo- differentiating HPCs of control (c). **(A, B)** The results of three independent experiments (mean ± SEM values) are shown; significance is *p <0.05; ns is for no significant. **(C)** Morphological analysis at day 21 of the differentiation and maturation of G and Mo differentiating HPCs treated with AR-C or SYRO, as compared to control (c) HPCs, and stained with May-Grünwald-Giemsa. One representative experiment out of three is shown.

To analyze the role of MCT1 and MCT4 during Mo and G proliferation and differentiation of HPCs, we inhibited their activity by using their respective inhibitor, AR-C and SYRO. AR-C and SYRO, used at 1 µM from day 0 (CD34^+^) and added every 2 days in culture, have no significant effect on cell growth of G and Mo differentiating HPCs (**Figure 2A**), except for a moderate effect of SYRO on cell growth of G differentiating HPCs, detectable only at late steps of G maturation. Cell cycle and differentiation of HPCs, as assessed by immunophenotypic analysis of differentiation-related markers, were not significantly affected by AR-C or SYRO treatment in G and Mo cultures (not shown) and we could not find any significant effect of these inhibitors on the clonogenetic capacity of G and Mo HPCs (**Figure 2B**). Morphology analysis of HPCs at day 21 of culture did not show any significant effect of AR-C and SYRO on G and Mo maturation (**Figure 2C**). Altogether, our data indicate that inactivation of MCT1 or MCT4 by using 1 µM of AR-C or SYRO, is without harmful effects on survival, proliferation and maturation of normal Mo and G differentiating HPCs.

Then, we analyzed MCT1 and MCT4 expression in several AML cell lines. MCT1 and MCT4 mRNAs are overexpressed in pro-monocytic U937 and promyelocytic NB4 leukemia cell lines, as compared to normal CD34^+^ HPCs and G and Mo differentiating HPCs (**Figures 1A, B** **)**. Although MCT1 and MCT4 expression levels differ between cell lines from different AML types, they are co-expressed in all leukemic cell lines that we have analyzed, excepted for the myelomonocytic MV4-11 cell line, negative for MCT4 expression (**Supplementary Figure 2A**). Differentiation of leukemic cells mimics the inverse expression of MCT1 and MCT4 observed during Mo and G differentiation of HPCs. MCT1 protein level decreases during vitamin D3- or TPA- induced Mo differentiation of U937 cells and during ATRA-induced G differentiation of NB4 cell lines, while MCT4 protein level increases (**Supplementary Figure 2B**). MCT1 and MCT4 expression level is not affected by ATRA treatment of NB4-R4 cells, highly resistant to retinoid-induced cytodifferentiation and maturation (**Supplementary Figure 2C**).

Altogether, our data show that MCT1 and 4 are inversely expressed during Mo and G proliferation and differentiation of normal and leukemic cells. MCT1 expression profile matches with CD147 expression (22), in line with a role for the MCT1-CD147 axis during normal and leukemic cell proliferation that may require a major uptake of lactate. Interestingly, differentiation-induced MCT4 expression that indicates an increased glycolytic activity in Mo and G differentiating cells, does not match with the profile of expression of its chaperone, CD147 (22).

### MCT4 Overexpression Is Associated With Poor Prognosis in AML

**Figure 3:**
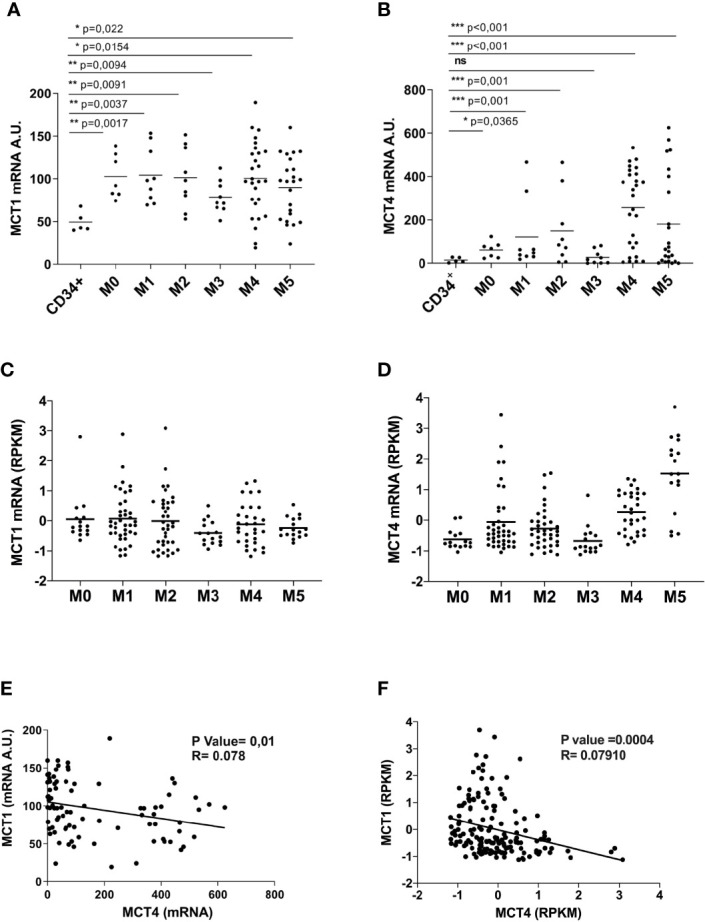
Overexpressed in AML, as compared to normal CD34^+^ HPCs, MCT1 and MCT4 mRNA expression is inversely correlated in AMLs. **(A, B)** qRT-PCR analysis of MCT1 and MCT4 mRNA expression in primary leukemic cells of AMLs pertaining from M0 to M5 subtypes of FAB classification, as compared to normal CD34^+^ HPCs. **(A, B)** The results of three independent experiments (mean ± SEM values) are shown; significance *, **, and *** are p <0.05, p <0.01, and p <0.001, respectively, ns is for no significant. **(C, D)** MCT1 and MCT4 mRNA expression data analysis from AML samples generated by TCGA Research Network. **(E, F)** Inverse correlation between MCT1 and MCT4 mRNA expression levels in AMLs, according data from: **(E)** our AML samples; **(F)** AML samples generated by TCGA Network. **(A, B, E)** AU is for arbitrary units. **(C, D, F)** RPKM is for Reads Per Kilobase of exon per Million mapped reads.

We analyzed MCT1/4 expression in primary leukemic blast cells from AML patients and found that MCT1 and 4 mRNAs are overexpressed in most of AML patients, as compared to normal CD34^+^ HPCs (**Figures 3A, B** **)**. However, MCT4 mRNA levels are higher than those of MCT1, suggesting a high and continued glycolytic activity of AML blasts (**Figure 3B**), particularly in AML pertaining to the M4 and M5 subtypes, as compared to CD34^+^ HPCs. These data were confirmed by the analysis of the TCGA Research network that provides genetic and clinical data from 200 AML patients (33) (**Figures 3C, D** **)**. Then, we found a significant inverse correlation between MCT1 and MCT4 mRNA expression levels in AMLs (**Figure 3E**), in line with TCGA data analysis (**Figure 3F**). We also found a significant positive correlation between MCT4 and CD147 mRNA expression in AMLs, whereas an inverse correlation was found between MCT1 and CD147 mRNAs in AMLs (**Supplementary Figure 3**).

**Figure 4:**
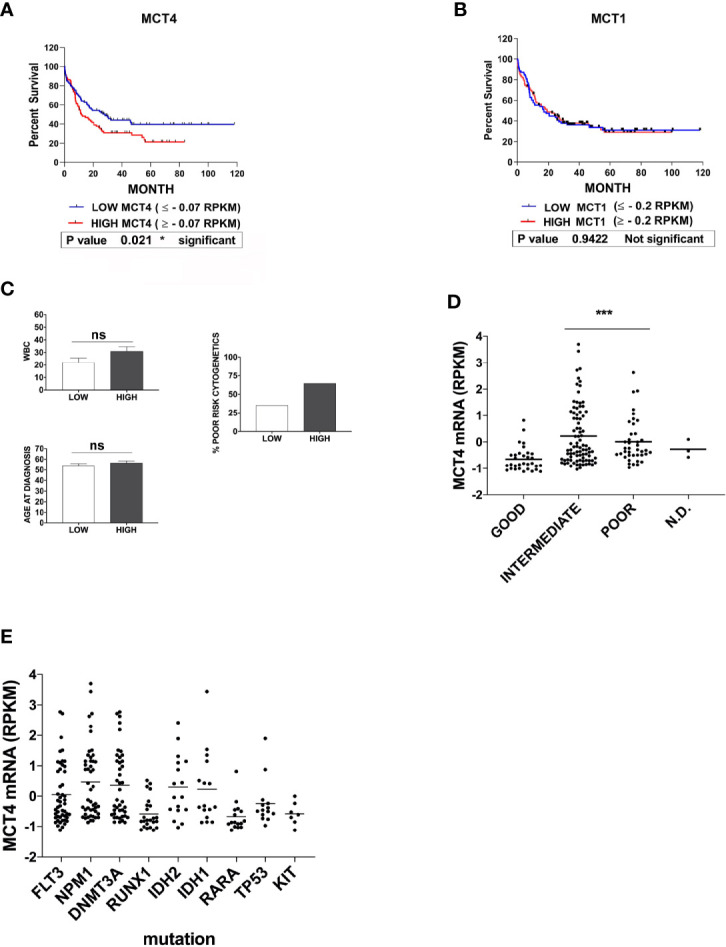
High expression of MCT4 is correlated with poor overall survival in AML patients, but not MCT1 expression. **(A)** Kaplan-Meier survival analysis in AMLs patients, based on MCT4 gene expression and stratified according to their MCT4 mRNA levels in two groups of patients : Low MCT4 (MCT4 ≤ -0.07 RPKM) and high MCT4 (MCT4 ≥ -0.07 RPKM), indicates that the kinetic of death is more rapid (within 80 months) among MCT4 high-patients, as compared with those with low MCT4 levels (p = 0.021). **(B)** Kaplan-Meier survival analysis in AMLs patients, based on MCT1 gene expression and stratified according to their MCT1 mRNA levels in two groups: Low (MCT1 ≤ -0.2 RPKM); High (MCT1 ≥ -0.2 RPKM), indicates that the kinetic of death is similar among MCT1 high-patients, as compared with those with low (p = 0.9422) MCT1 levels. **(A, B)** P-value is calculated by using the log-rank test. **(A, B)** RPKM is for Reads Per Kilobase of exon per Million mapped reads. **(C)** White blood count (WBC) number, at age diagnosis and proportion of patients with poor cytogenetics were comparable in both AML subgroups subdivided according to MCT4 expression level, ns is for no significant. **(D)** MCT4 mRNA levels were analyzed in AMLs stratified into three risk groups, poor, intermediate, and good/favorable, according to the European LeukemiaNet (ELN) risk classification. Significance *** is p <0.001. **(E)** Relationship between the most recurrent gene mutations observed in all AMLs and the level of MCT4 mRNA expression, according to TCGA dataset.

To evaluate a possible correlation between MCT1 or MCT4 expression level and overall survival of AML patients, we stratified all AMLs into three risk group according to the European LeukemiaNet (ELN) risk classification (33) and classified MCT1/4 expression levels as: low (MCT1 ≤ -0.2; MCT4 ≤ -0.07); high (MCT1≥ -0.2; MCT4 ≥ -0.07). During 120 months, a significant correlation was detected between MCT4 levels and overall survival of AML patients (p = 0,021), but not for MCT1 (p = 0,942) (**Figures 4A, B** **)**. Death during the first 80 months of follow-up was observed more frequently in cases with high MCT4 levels, as compared to those with low MCT4 levels (p = 0,0385) (**Figure 4A**). The two-AML subgroups, with low and high MCT4 levels, show comparable age and white blood count (WBC) number at diagnosis (**Figure 4C**). Lower MCT4 mRNA levels were observed in favorable-risk AMLs, compared to those observed in poor- and intermediate -risk group AMLs (**Figure 4D**), without any significant relationship between the recurrent gene mutations (**Figure 4E**) observed in AML and the level of MCT4 expression.

Altogether, our data indicate that only MCT4 high level is associated with poor survival in AML patients, as previously described for CD147 (22).

### Inhibition of MCT1 and MCT4 by AR-C or SYRO Treatment Is Selective and Alters Lactate Metabolism of Leukemic Cells

**Figure 5:**
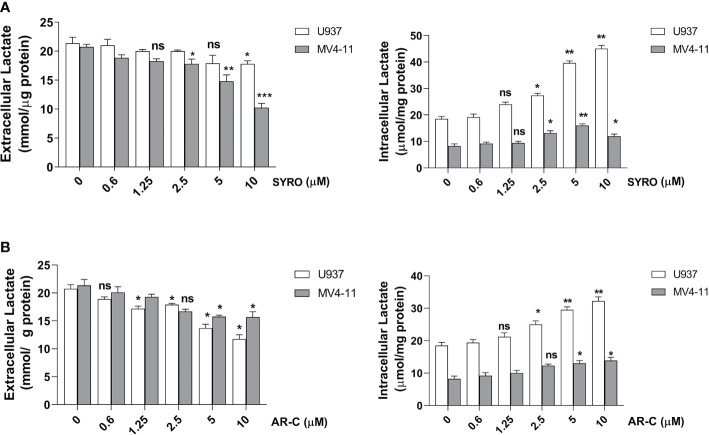
Dose response analysis of SYRO and AR-C treatment of U937 and MV4-11 cells. **(A)** Lactate Glo assays were performed to analyze intracellular and extracellular lactate levels in U937 and MV4-11 leukemic cells treated 2 h by SYRO, as compared to untreated cells of control (0). **(B)** Lactate Glo assays were performed to analyze intracellular and extracellular lactate levels in U937 and MV4-11 cells treated 2 h by AR-C, as compared to untreated cells of control (0). **(A, B)** The results of three independent experiments (mean ± SEM values) are shown; significance is *p <0.05; **p <0.01; ***p <0.001; ns is for not significant.

To understand whether MCT1 and MCT4 overexpression translates into increased lactate flux capacity of leukemic cells, we inhibited their activity by using AR-C and SYRO and analyzed the metabolism of leukemic cells treated with AR-C or SYRO, as compared to AC-73-treated cells and untreated control cells. By using Lactate-Glo assays, we evaluated intra- and extracellular lactate levels changes in leukemic cells treated with increasing doses of AR-C or SYRO, as compared to untreated cells (0), and showed that inhibition of MCT1/4 activity is dose dependent of AR-C and SYRO treatment in U937 and MV4-11 cells (**Figure 5**).

**Figure 6:**
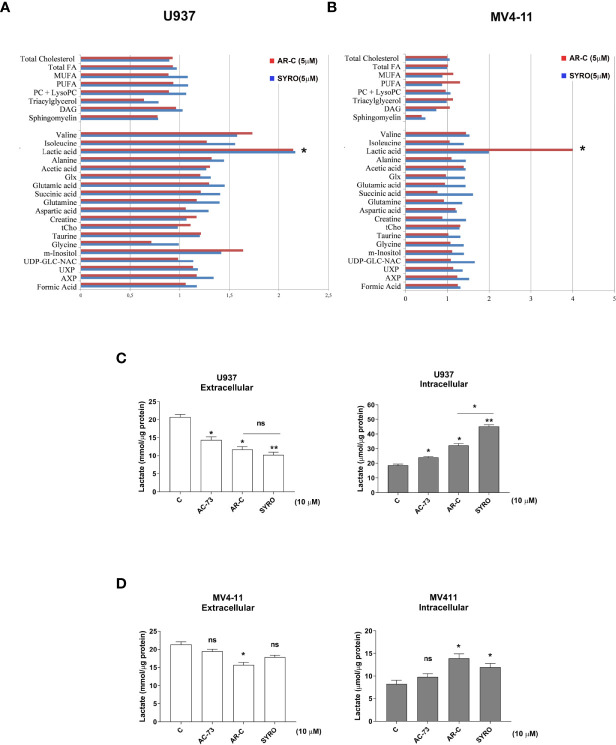
AR-C and SYRO are selective inhibitors of lactate metabolism in leukemic cells. **(A, B)** Intracellular/extracellular metabolome analysis by NMR spectroscopy in U937 and MV4-11 cells treated 24 h with AR-C and SYRO used at 5 µM, normalized to untreated cells. Fold increase of intracellular aqueous and lipid metabolites in SYRO- and AR-C-treated U937 (n = 3) and MV4-11 cells (n = 2) relative to untreated cells (reference value = 1) is shown. Lipid metabolites: DAG, Diacylglycerols; FA, Fat acids; MUFA, Monounsaturated FA; PC, Phosphatidylcholine; LysoPC, Lyso-phosphosphatidylcholine; PUFA, Polyunsaturated FA. Aqueous metabolites: AXP, (AMP+ADP+ATP); Glx, Glutamic acid + Glutamine; tCho, total choline = (free choline + glycerophosphocholine + phosphocholine); UDP-GLC-NAC, Uridine diphosphate N-acetylglucosamine; UXP, (UMP+UDP+UTP). **(C, D)** Lactate Glo assays were performed to analyze intra and extracellular lactate levels in U937 and MV4-11 cells treated 2 h with 10 µM of AR-C or SYRO, as compared to U937 and MV4-11 leukemic cells treated 2 h with AC-73 (10 µM) and to untreated cells of control (c). The results of three independent experiments (mean ± SEM values) are shown; significance * and ** are p <0.05 and p <0.01, respectively; ns is for not significant.

Then, we carried out aqueous and lipid intracellular metabolomics analysis by NMR spectroscopy in the dual MCT1 and 4 expressing U937 cells and in the MCT1 expressing MV4-11 cells treated 24 h with AR-C and SYRO used at 5 µM (dose with NMR-detectable metabolic changes and non-toxic), as compared to untreated cells. We observed an average increase (range between 2–4 fold) in the accumulation of intracellular levels of lactate in both SYRO and AR-C treated U937 (P < 0.05; n = 3) and MV4-11 (n = 2) cells, as shown by NMR (**Figures 6A, B** **)** and metabolites quantification, as compared to untreated cells (**Table 1**). We did not find any significant changes in other intracellular NMR–detectable metabolites involved in different biochemical pathways, such indicating a selective inhibition for lactate metabolism of these drugs.

**Table 1:** Absolute and relative quantification of intracellular aqueous metabolites in untreated cells (CTRL) and SYRO- and AR-C-treated U937 and MV4-11 cells.

Metabolites	Absolute Quantification (nmoles/106 cells)						Fold change vs CTRL			
		U937			MV4-11		U937		MV4-11	
	CTRL	SYRO	AR-C	CTRL	SYRO	AR-C	SYRO	AR-C	SYRO	AR-C
Formic acid	4.60 ± 1.44	6.19 ± 1.28	5.14 ± 2.74	3.60 ± 2.69	5.83 ± 4.40	5.83 ± 4.71	1.2 ± 0.2	1.1 ± 0.2	1.3 ± 0.4	1.3 ± -0.4
AXP	4.83 ± 2.05	5.15 ± 1.53	4.74 ± 1.42	2.60 ± 0.55	4.14 ± 0.34	2.82 ± 0.13	1.3 ± 0.4	1.2 ± 0.5	1.5 ± 0.1	1.2 ± 0.1
UXP	5.46 ± 3.63	5.21 ± 4.38	4.68 ± 4.04	2.51 ± 2.01	2.89 ± 2.47	1.62 ± 1.24	1.2 ± 0.1	1.1 ± 0.5	1.4 ± 0.1	1.1 ± 0.2
UDP-GLC-NAC	14.71 ± 7.22	13.23 ± 6.14	11.54 ± 6.29	6.98 ± 2.25	10.92 ± 2.18	6.15 ± 0.19	1.1 ± 0.4	1.0 ± 0.5	1.7 ± 0.2	1.1 ± 0.2
myo-Inositol	3.11 ± 1.12	4.14 ± 1.17	2.78 ± 2.89	9.92 ± 3.99	14.43 ± 4.67	8.62 ± 1.42	1.4 ± 0.3	1.6 ± 0.1	1.4 ± 0.1	1.1 ± 0.0
Glycine	6.24 ± 4.33	5.66 ± 4.05	4.50 ± 3.36	2.64 ± 1.22	4.69 ± 0.66	2.39 ± 0.88	1.0 ± 0.1	0.7 ± 0.3	1.4 ± 0.1	1.1 ± 0.1
Taurine	9.16 ± 3.69	10.46 ± 3.38	9.85 ± 4.21	9.18 ± 2.39	13.76 ± 0.24	8.42 ± 1.57	1.2 ± 0.3	1.2 ± 0.4	1.3 ± 0.0	1.0 ± 0.0
Total Choline	12.64 ± 1.77	12.90 ± 0.33	12.91 ± 2.08	3.99 ± 1.86	6.85 ± 3.42	3.50 ± 1.75	1.0 ± 0.1	1.1 ± 0.1	1.3 ± 0.6	1.3 ± 0.7
Glycerophosphocholine	0.18 ± 0.15	0.15 ± 0.21	bd	0.43 ± 0.49	0.61 ± 0.45	0.20 ± 0.20	1.1 ± 1.6	bd	0.8 ± 0.3	0.4 ± 0.2
Phosphocholine	10.01 ± 4.82	10.45 ± 3.80	10.29 ± 3.04	2.66 ± 1.84	3.44 ± 2.51	1.98 ± 1.61	1.2 ± 0.4	1.4 ± 0.4	1.3 ± 0.0	0.9 ± 0.4
Choline	0.63 ± 1.01	0.13 ± 0.18	0.35 ± 0.27	0.18 ± 0.16	0.82 ± 0.30	0.14 ± 0.12	0.9 ± 0.3	2.1 ± 2.9	3.9 ± 2.4	1.1 ± -0.4
Creatine	1.47 ± 0.60	1.52 ± 0.80	1.45 ± 0.58	4.62 ± 6.50	9.84 ± 8.46	4.89 ± 4.13	1.1 ± 0.1	1.2 ± 0.5	1.5 ± 0.1	0.9 ± 0.1
Aspartic acid	2.81 ± 1.85	2.34 ± 1.56	1.79 ± 0.54	2.42 ± 1.16	2.98 ± 1.53	1.96 ± 0.01	1.3 ± 0.2	1.1 ± 0.8	1.2 ± 0.0	1.2 ± 0.4
Succinic acid	0.73 ± 0.27	0.92 ± 0.50	0.68 ± 0.13	0.61 ± 0.32	1.26 ± 0.13	0.41 ± 0.19	1.4 ± 0.5	1.2 ± 0.6	1.6 ± 0.1	0.8 ± 0.1
Glutamic acid	10.83 ± 4.52	14.16 ± 7.78	12.31 ± 4.99	10.02 ± 5.34	15.68 ± 7.71	7.07 ± 3.38	1.5 ± 0.2	1.3 ± 0.3	1.4 ± 0.0	0.9 ± 0.3
Glutamine	7.22 ± 4.17	7.56 ± 3.60	5.94 ± 1.68	3.47 ± 0.75	5.28 ± 0.14	2.94 ± 0.61	1.4 ± 0.2	1.2 ± 0.5	1.4 ± 0.0	0.9 ± 0.0
Glx	26.54 ± 11.45	29.99 ± 13.68	26.09 ± 9.53	18.77 ± 6.61	29.10 ± 8.92	14.53 ± 1.72	1.3 ± 0.2	1.2 ± 0.4	1.4 ± 0.0	1.0 ± 0.1
Acetic acid	1.48 ± 1.21	1.26 ± 0.88	1.68 ± 1.36	0.68 ± 0.30	0.81 ± 0.33	0.88 ± 0.35	1.3 ± 0.3	1.3 ± 0.3	1.4 ± 0.1	1.4 ± 0.1
Alanine	4.84 ± 2.46	6.01 ± 2.57	5.87 ± 2.78	2.11 ± 1.31	3.38 ± 1.20	1.49	1.4 ± 0.4	1.3 ± 0.5	1.4 ± 0.2	1.1 ± 0.0
Lactic acid	16.81 ± 12.44	34.64 ± 25.99	30.16 ± 17.47	7.97 ± 2.97	19.31 ± 4.94	38.95 ± 32.25	2.2 ± 0.7	2.1 ± 1.3	2.0 ± 0.3	4.0 ± 2.6
Isoleucine	0.50 ± 0.52	0.42 ± 0.50	0.64 ± 0.32	0.24 ± 0.21	0.27 ± 0.27	0.39 ± 0.08	1.6 ± 0.1	1.3 ± 0.7	1.4 ± 0.7	1.1 ± 0.2
Valine	0.67 ± 0.56	0.98 ± 0.67	1.05 ± 0.69	0.57	0.87 ± 0.44	0.83 ± 0.41	1.6 ± 0.3	1.7 ± 0.4	1.5 ± 0.8	1.5 ± 0.7

bd, below detection; Metabolites: AXP, (AMP+ADP+ATP); Glx, Glutamic acid + Glutamine; UXP, (UMP+UDP+UTP); UDP-GLC-NAC, Uridine diphosphate N-acetylglucosamine.

We also quantified using Lactate-Glo assays, intra- and extra-cellular lactate levels in U937 and MV4-11 cells treated in short time window (within 2 h) with a high concentration (10 µM) of AR-C, SYRO or AC-73 (**Figures 6C, D** **)**. AR-C and SYRO induce a significant accumulation of intracellular lactate and concomitant reduced extracellular lactate in U937 cells, with a major efficiency for SYRO targeting MCT4 in these cells **(** **Figure 6C**). In MV4-11 cells, AR-C and SYRO induce a significant but modest accumulation of intracellular lactate in these cells while concomitant reduced extracellular lactate level is detected only in AR-C-treated MV4-11 cells (**Figure 6D**), such indicating a defect in lactate export of these cells lacking MCT4 and that MCT1, as a bidirectional transporter of lactate, may supply. It is of interest to note the effect of the inhibitor of CD147, AC-73, that induces a lower but significant accumulation of intracellular lactate in U937 cells, but not in MV4-11 cells, as compared to AR-C and SYRO treatment in these cells (**Figures 6C, D** **)**. AC-73 that inhibits CD147 dimerization (22, 26), disturbs CD147-MCT4 co-binding and function in membrane. As expected, combined treatment of AC-73 with AR-C or SYRO induces a metabolic catastrophe in leukemic cells (not shown).

Altogether, our data show that AR-C and SYRO by inactivating their selective target, MCT1 and MCT4 respectively, inhibit glycolysis in leukemic cells and cause lactate accumulation in AML cells. Although AR-C and SYRO have a different impact on the rate of import and export of lactate in leukemic cells, SYRO appears more efficient in blocking the lactate flux capacity of leukemic cells by predominantly inhibiting lactate export *via* MCT4.

### Selective Inhibition of MCT1 or MCT4 Inhibits Leukemic Cell Proliferation by Activating Two Different Cell-Death Related Pathways, Autophagy and Necrosis

**Figure 7:**
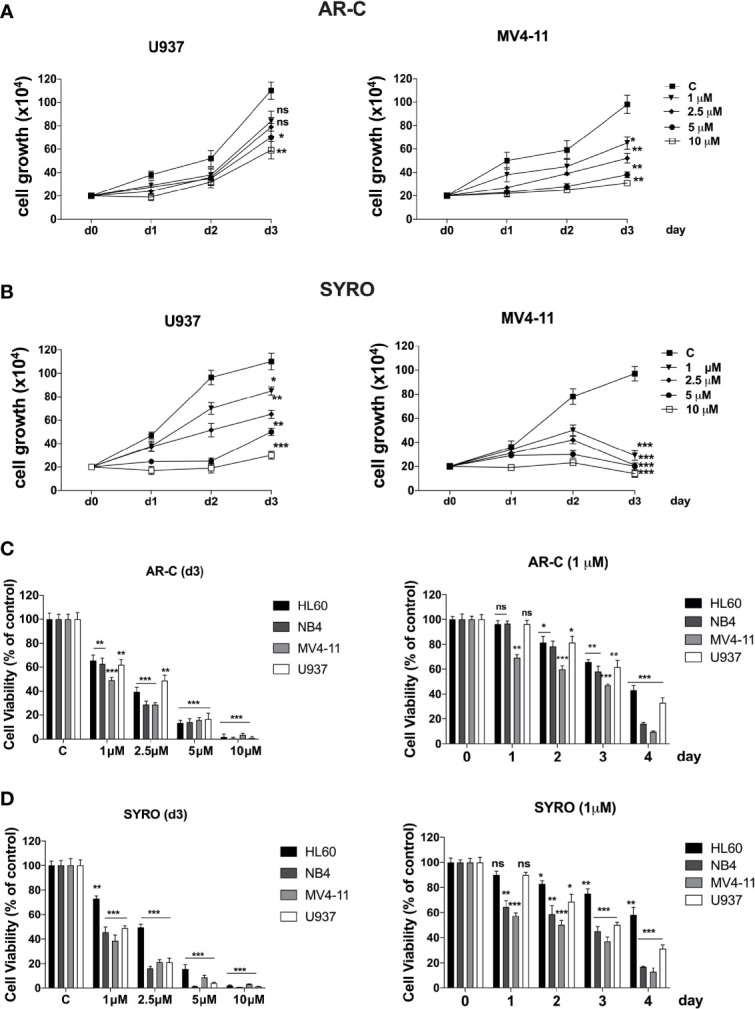
Effects of AR-C and SYRO on cell growth and viability of AML cell lines. **(A, B)** Dose response analysis of AR-C **(A)** and SYRO **(B)** treatment on U937 and MV4-11 leukemic cell growth, as compared to control cells (c). **(C)** Cell viability assays on leukemic cells treated: (left panel) with AR-C used at different concentrations for 3 days; (right panel) at different times with 1 µM AR-C, as compared to control cells (day 0). **(D)** Cell viability assays on leukemic cells treated: (left panel) with SYRO used at different concentrations for 3 days; (right panel) at different times with 1 µM SYRO, as compared to control cells (day 0). **(C, D)** Viability is presented as percentage viable cell relative to control. **(A–D)** The results of three independent experiments (mean ± SEM values) are shown; significance is *p <0.05; **p <0.01; ***p <0.001; ns is for not significant.

**Figure 8:**
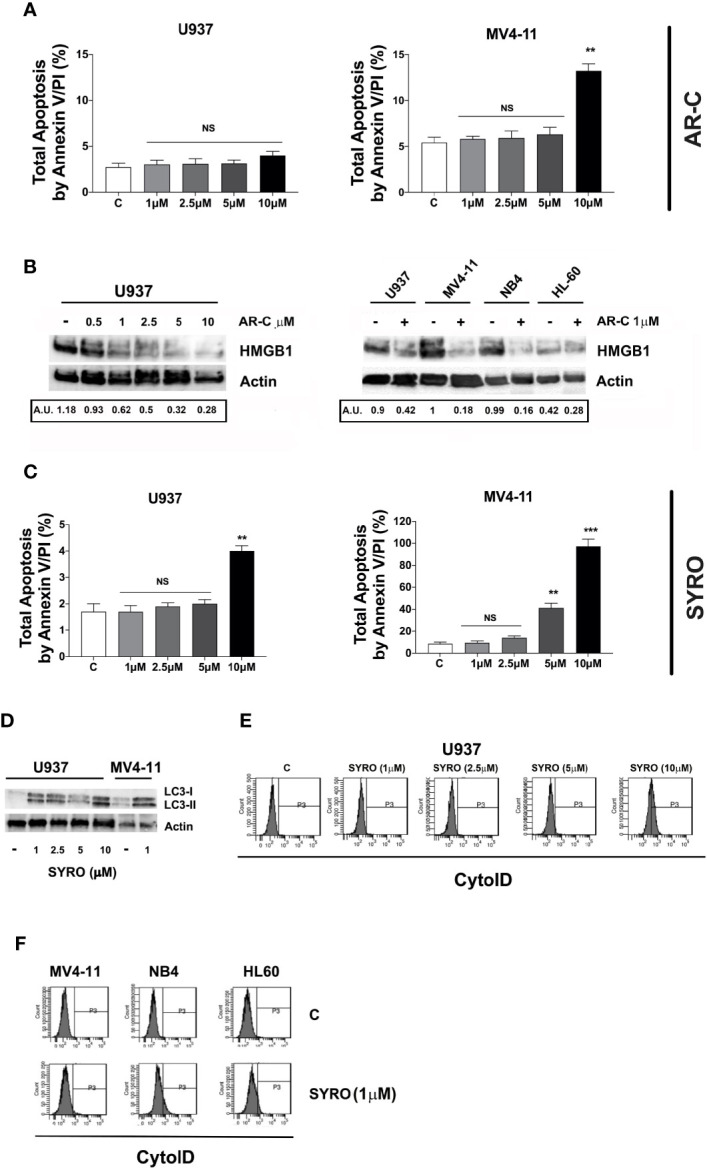
Effects of AR-C and SYRO on apoptosis and on the related cell-death pathways, necrosis, and autophagy. **(A)** Dose response analysis of AR-C treatment performed for 3 days on leukemic cell apoptosis, as compared to control (c) leukemic cell. **(B)** Western blot analysis of the necrosis related HMGB1 protein in U937 cells treated with AR-C used at different concentrations (left panel) and used at 1 µM in AML cell lines (right panel), as compared to untreated cells of control (–) cells. Quantification of HMGB1 proteins by densitometry analysis is indicated (AU is for arbitrary units). **(C)** Dose response analysis of SYRO treatment performed for 3 days on leukemic cell apoptosis, as compared to control (c) leukemic cells. **(D)** Western blot analysis of the autophagy related protein LC3 and its conversion from LC3-I to LC3-II form in U937 cells treated with SYRO used at different concentrations, and in MV4-11 cells treated with 1 µM SYRO, as compared to untreated leukemic cells (–). **(E)** Dose response analysis of SYRO treatment on the autophagy flux in U937 leukemic cells, as compared to control cells (c). **(F)** Induction of autophagy by SYRO treatment used at 1 µM for 3 days in several AML cell lines, as compared to control cells (c). **(A, C)** The results of three independent experiments (mean ± SEM values) are shown; significance is **p <0.01; ***p <0.001; NS is for not significant. Total apoptosis by annexin V/PI (%) detected by using flow cytometric apoptotic assays, is indicated. **(B, D** left panel**)** One representative western blot experiment out of three is shown; Actin is an internal control. **(E, F)** One representative experiment out of three is shown.

Then, we analyzed the impact of a chronic administration of AR-C and SYRO on leukemic cell growth, apoptosis, and viability of AML cell lines. Leukemic cells were treated from 1 to 3 days, with various AR-C or SYRO doses (1.0, 2.5, 5.0, and 10 µM). We observed a time- and dose-dependent effect of both AR-C and SYRO on leukemic cell growth, with growth inhibition of all leukemic cell lines treated (**Figures 7A, B**, **Supplementary Figures 4A, B** **)**. AR-C and SYRO treatment have no significant effect on cell cycle distribution in the leukemic cell lines tested, as compared to control cells (not shown), suggesting that both AR-C and SYRO decrease the cell growth rate but do not inhibit the progression of cells in specific cell cycle steps. Cell viability assays were performed to assess leukemic cell survival and sensitivity to AR-C and SYRO treatment, as compared to untreated leukemic cells. We found a significant time- and dose- dependent decrease of leukemic cell viability in all leukemic cell lines treated with AR-C or SYRO, as compared to untreated cells (**Figures 7C, D** **)**. But we also observed a greater efficiency of SYRO, as compared to AR-C treatment, in both inhibiting cell growth (**Figures 7A, B**, **Supplementary Figures 4A, B** **)** and cell viability (**Figures 7C, D** **)**, in line with our data indicating a major efficiency of SYRO to inhibit lactate metabolism of dual MCT1/MCT4-expressing U937 cells, as compared to AR-C (**Figure 6C**). However, whether we cannot exclude that the different sensitivities to AR-C and SYRO manifested by the different leukemic cell lines, may be also related to the specific molecular alteration of these cell lines (*PML/RARA*, *FLT3*-ITD and *RUNX1/RUNX1T1*), we couldn’t detect any significant effect on apoptosis, except when leukemic cells were treated with high concentration (10 µM) of AR-C or SYRO (**Figures 8A, C**; **Supplementary Figures 4C, D** **)**. Since low doses of these drugs inhibit leukemic cell proliferation, but do not cause cell death *via* apoptosis or cell cycle arrest, we investigated which cell-death pathway may be activated by AR-C and SYRO treatment in leukemic cells. Flow cytometry analysis in live leukemic cells could not detect any induction of autophagy in leukemic cells treated with AR-C, as compared to untreated cells (not shown). Then, we analyzed whether AR-C could induce necrosis in leukemic cells by biochemical detection of high mobility group box 1 (HMGB1) release, HMGB1 being a factor that starts and promotes inflammation when it is released during necrosis (34). Western blot analysis showed a dose-dependent effect during 2 days of AR-C treatment associated with the decrease of HMGB1 protein level in AR-C-treated U937 cells, as compared to untreated (-) cells (**Figure 8B**, left panel), such indicating an increase of HMGB1 released from AR-C-treated U937 cells, as compared to U937 untreated cells. A decrease of HMGB1 protein level was also found in other AML cells lines treated with AR-C (+), as compared to untreated (-) cells (**Figure 8B**, right panel), thus indicating that AR-C induces necrosis in leukemic cells.

We also monitored the autophagy flux in live leukemic cells treated 2 days with SYRO, as compared to untreated control cells. We found that SYRO induces autophagy in leukemic cells (**Figure 8D**), in a dose-dependent way, as shown in U937 cells (**Figure 8E**). Then, we assessed the effect of SYRO on the level of both the autophagic indicator LC3 (22, 35) and HMGB1 for necrosis by Western blotting in U937 and MV4-11 cells. Our results showed a dose-dependent effect of SYRO on the increase of LC3-II/LC3-I ratio in U937 cells, as compared to control (-) cells, in MV4-11 and others AML cells (**Figure 8F**), confirming the induction of autophagy by SYRO in leukemic cells. We couldn’t detect any significant decrease of HMGB1 protein level in SYRO-treated leukemic cells, as compared to untreated cells (not shown). Altogether, our data showed that AR-C and SYRO by inhibiting MCT1 and MCT4 respectively, inhibit lactate metabolism and leukemic cell proliferation by inducing two different cell-death related pathways in AML cells i.e., necrosis for AR-C; autophagy for SYRO, a dual inhibitor of MCT1 and 4 but with a major affinity for MCT4 involved in lactate export in leukemic cells overexpressing this transporter.

### AR-C and SYRO Potentiate Cytotoxicity of Ara-C in AML Cells

**Figure 9:**
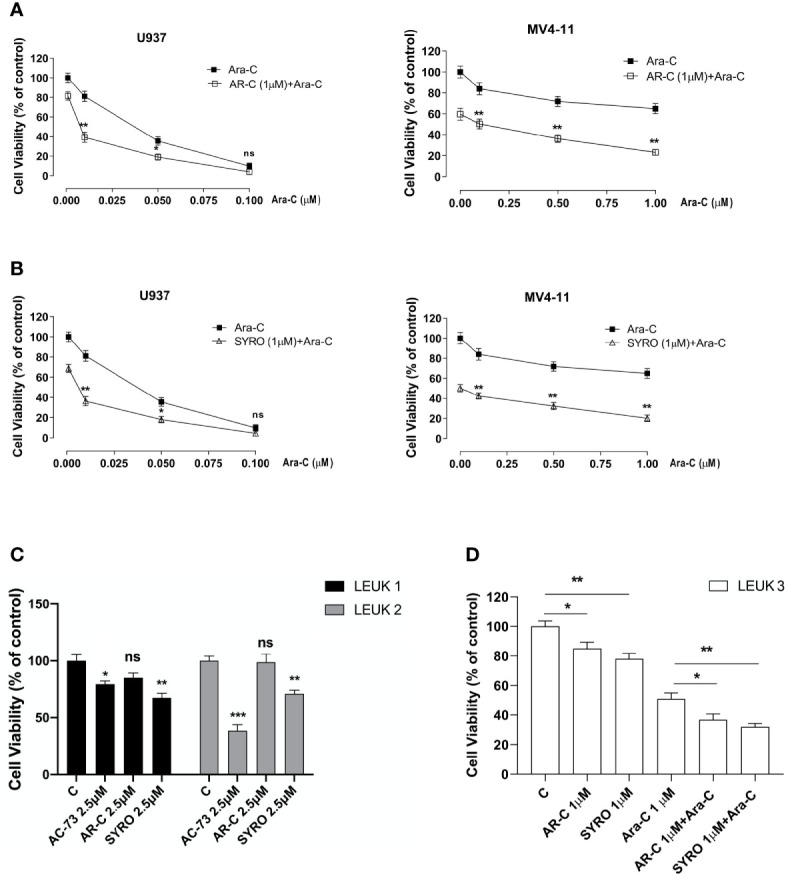
AR-C and SYRO by inhibiting lactate metabolism, decrease cell viability and potentiated cytotoxicity of Ara-C in AML cells. **(A)** Cell viability assays on U937 and MV4-11 leukemic cells, treated 24 h with AR-C used alone (1 µM), then in combination with Ara-C (AR-C (1µM) + Ara-C), as compared to treatment with single drug, AR-C (AR-C + 0.00 µM Ara-C). **(B)** Cell viability assays on U937 and MV4-11 leukemic cells, treated 24 h with SYRO used alone (1 µM), then in combination with Ara-C (SYRO (1µM) + Ara-C), as compared to treatment with single drug, SYRO (SYRO + 0.00 µM Ara-C). **(C)** Cell viability assays were performed on leukemic blasts obtained from 2 AML patients LEUK1 and LEUK2, treated *in vitro* by single drug AC-73, AR-C or SYRO used at 2.5 µM, as compared to control (c) leukemic blasts. **(D)** Cell viability assays performed on leukemic blasts of one AML patient LEUK 3, treated *in vitro* by AR-C or SYRO used at low dosage (1 µM) in combination with Ara-C (1µM), as compared to single treatment with AR-C, SYRO and Ara-C (1 µM) and to control (c) leukemic blasts. **(A–D)** The results of three independent experiments (mean ± SEM values) are shown; significance is *p <0.05; **p <0.01; ***p <0.001; ns is for no significant.

To evaluate the effect of AR-C and SYRO in combination with Ara-C, a standard drug used for AML treatment, AML cell lines were initially treated for 24 h with a low concentration (1 µM) of AR-C **(** **Figure 9A**) or SYRO (**Figure 9B**) and then co-treated for 48 h with Ara-C at different concentrations; from 0.025 to 0.1 µM for U937 cells (**Figures 9A, B**, left panels) and from 0.25 to 1.00 µM for MV4-11 cells (**Figures 9A, B**, right panels). Cell viability assays were performed to assess leukemic cell survival and sensitivity to AR-C and SYRO treatment used in combination with Ara-C, as compared to single drug treatment (**Figures 9A, B**: 0.00 µM). We observed a significant decrease of leukemic cell viability of both U937 and MV4-11 leukemic cell lines after AR-C or SYRO treatment in combination with Ara-C, as compared to AR-C, SYRO or Ara-C used alone (**Figures 9A, B** **)**.

We also evaluated the effects of AR-C and SYRO used alone at 2.5 or 1 µM, on primary AML blasts obtained from 3 AML patients (LEUK 1, 2, and 3), as compared to AC-73 (2.5 µM) (**Figure 9C**) or to low dose of Ara-C (1 µM) (**Figure 9D**) treatment and to untreated control cells (C). In LEUK 1 and LEUK2, SYRO is significantly more efficient than AR-C, to decrease leukemic cells viability (**Figure 9C**), both AML patients expressing high level of MCT4 (not shown). AC-73, as previously described (22), is an efficient inhibitor of leukemic blasts viability (**Figure 8C**). In LEUK 3, that we treated with lower dosage (1 µM) of drugs, we always found a major efficiency of SYRO than AR-C treatment, in inhibiting leukemic blasts viability, but we also found that combination of low dose AR-C or SYRO (1 µM) with Ara-C (1µM) decreased more efficiently leukemic blasts viability, as compared to single AR-C, SYRO or Ara-C treatment (**Figure 9D**).

Overall, our data indicate that AR-C and in particular, SYRO, by selectively inactivating MCT1 and MCT4 function, decrease leukemic cells viability and increase the sensitivity of leukemic cells to Ara-C.

## Discussion

MCT1 and MCT4 overexpression and co-overexpression with CD147, are considered a hallmark of cancer (1–6), indicating the inactivation of these transporters with specific inhibitors or by targeting their chaperone CD147 (11–14), as a promising strategy for cancer therapy (1–5, 8, 22, 26–31). However, the activity of MCT1 and MCT4 removing the excessive levels of lactate that are produced by cancer cells, has not been extensively studied in AML cells. Lactic acidosis is a rare complication in acute leukemia (36, 37) and metabolism in AML cells is primarily dependent on OXPHOS, the metabolic pathway that takes place inside mitochondria (17, 18, 24, 25). However, through clonal evolution, leukemic hematopoiesis in AML constantly adapts to environmental conditions and gains metabolic plasticity, leading to rapidly outcompete normal hematopoietic cells (25, 38, 39) to survive and proliferate. In this context, a dependence on glycolysis in aerobic conditions has also been described in acute leukemia (39, 40). Then, understanding whether MCT1 and MCT4 represent new therapeutic targets and whether their inhibitors AR-C and SYRO can be used in leukemia therapy, may reveal an alternative treatment strategy in patients with AML.

In our study, we found that MCT1 and 4 are inversely expressed i.e., MCT1 expression decreases while MCT4 increases, during Mo and G proliferation and differentiation of normal and leukemic cells. MCT1 expression profile matches with CD147 expression (22), in line with a role for the MCT1-CD147 axis during normal and leukemic cell proliferation that requires a major uptake of lactate. Instead, differentiation-induced MCT4 expression indicates an increased glycolytic activity of Mo and G differentiating HPCs, particularly significant in Mo cells and in line with the increased expression of MCT4 described in macrophages and essential to a fully activated inflammatory response (32). Surprisingly, MCT4 expression profile does not match that observed for its chaperone CD147 (22). However, the level of CD147 highly glycosylated protein should be sufficient to exert its role of chaperone to transport MCT4 at the plasma membrane where they stabilize mutually and where MCT4 acts to export lactate (22, 41).

Then, we found that MCT1 and MCT4 are overexpressed in leukemic cell lines as compared to normal CD34+ HPCs. However, Mo- and G- induced differentiation of leukemic cells restores MCT1 and MCT4 expression pathways observed in Mo and G lineage cells. Notably, we also found that MCT1 and 4 are co-overexpressed in the large majority of primary leukemic blasts analyzed, as compared to normal CD34^+^ HPCs, and in all FAB AML subtypes. These findings were corroborated by the analysis of the MCT1 and MCT4 expression in AML patients by TCGA data set. We found an inverse correlation between MCT1 and 4 mRNA expression levels in blasts of AML patients and showed that only MCT4 high mRNA level is correlated with poor prognosis in AML patients, as previously described for CD147 (22), thus indicating MCT4 as a potential therapeutic target in AML. Elevated levels of MCT4 were not associated with recurrent AML genetic alterations, such as *NPM1*, *FLT3-ITD*, *DNMT3A*, and *PML-RARA*.

Then, we analyzed the effects of MCT1 and MCT4 inhibition in AML cell lines and primary leukemic blasts, by using AR-C, a potent inhibitor of MCT1, with an analogue AZD3965 currently tested in clinical trials for several types of cancer (42), and SYRO, an antihypertensive agent that was found to potentiate the anticancer effects of the antidiabetic agent metformin and described as a dual inhibitor of MCT1 and 4 but with a major affinity for MCT4 (8). We found that low doses of AR-C and SYRO, by exerting a selective inhibition of lactate metabolism in leukemic cells, inhibit leukemic cell proliferation but do not cause cell death *via* apoptosis or cell cycle arrest. However, AR-C and SYRO activate two different cell-death related pathways i.e., necrosis for AR-C treatment and autophagy for SYRO, as previously observed for the inhibitor of CD147, AC-73 (22). Whether necrosis is considered as an accidental and uncontrolled cell death, a concept currently under revision (34), autophagy is a conserved and physiologically regulated cellular degradation pathway essential for hematopoietic stem cell maintenance, resistance to stress, survival and differentiation, a complex biological machinery deregulated in AMLs (35, 43). As MCT1 and MCT4 are expressed in normal HPCs, we also showed that a low dose of AR-C and SYRO induces a slight inhibition of cell proliferation, without significantly affecting differentiation of CD34^+^ HPCs. Then, we validated the potential therapeutic activity of AR-C and SYRO in AML cells, by using AR-C and SYRO in combination, or not, with conventional anti-leukemia treatment and as compared to AC-73 treatment (22). We showed that the anti-proliferative effect of AR-C and SYRO, used at low concentration, is sufficient to enhance the sensitivity of leukemic cells and primary AML blasts to the chemotherapeutic agent Ara-C, reducing the concentration of this drug required for its anti-neoplastic activity. Although SYRO is a potent inhibitor of the lactate transporters MCT1 and MCT4, we cannot exclude a possible off-target effect of this inhibitor. However, SYRO treatment appears more efficient than AR-C, as anti-proliferative drug and inducer of autophagy in leukemic cells overexpressing MCT4.

A recent study showed that VEGF regulates MCT1 expression in AML cells and through this mechanism adapts the metabolic fitness of leukemic cells to the microenvironment (44). Particularly, MCT1 was shown to mediate lactate uptake in leukemic cells to sustain OXPHOS (44). Our study complements these observations and suggests a relevant functional role for MCT4 in AML cells as a metabolic regulator; the elevated activity of MCT4 in AML cells is required for their survival and proliferation, as suggested by functional experiments using MCT4 inhibitors. Our observations are also in line with a recent study by Man et al. (45) showing that increased MCT4 expression observed in AML cells determines an intracellular alkalization through lactate extrusion, gaining a growth advantage without dependence on signaling pathways (45).

Numerous studies have shown remarkable differences between the metabolism of normal hematopoietic and leukemic stem cells and described the metabolic plasticity of AML cells that contributes to leukemic cell chemoresistance (38, 46, 47). Then, metabolism manipulation may be used to preferentially compromise leukemia cell growth but not normal hematopoiesis (17, 38, 40, 46, 47) and metabolic pathways can be exploited to repurpose drugs, such as syrosingopine. Altogether, we showed that pharmacological inactivation of MCT1 or MCT4 inhibits the proliferation of leukemic cells *in vitro*, thus indicating that targeting lactate metabolism in AML may be an opportunity for novel treatment strategies in AMLs. In particular, our study points to MCT4 as a potential therapeutic target in AML patients and to its inhibitor SYRO as a new anti-proliferative drug and inducer of autophagy, to be used in combination with conventional chemotherapeutic agents in AML treatment.

## Data Availability Statement

The original contributions presented in the study are included in the article/**Supplementary Material**, further inquiries can be directed to the corresponding author.

## Ethics Statement

For human cord blood from healthy donors: approval by local ethical committees of Istituto Superiore di Sanità, Rome (file number # 171639). For leukemic blasts from bone marrow obtained from patients with newly diagnosed AML: approval by local ethical committees of Tor Vergata University (file number RS 34.20 del 26/02/2020 Fondazione PTV Policlinico Tor Vergata, Rome). The patients/participants provided their written informed consent to participate in this study. 

## Author Contributions

ES participated in design of the study, performed experiments, and analyzed data. IS performed experiments and analyzed data. MQ, EP, and GC performed experiments. LP performed flow cytometry analysis. EI, MC, and MP performed NMR spectroscopy and metabolome analysis. TO and MV provided leukemic blasts from patients and contributed to manuscript editing. UT analyzed data and revised the paper. CL conceptualized the study and designed the research strategy, analyzed data, and wrote the manuscript. All authors contributed to the article and approved the submitted version. 

## Funding

CL was supported by Istituto Superiore di Sanità (Rome, Italy). MV was supported by PRIN (MIUR, Italy) and by AIRC 5x1000 call “Metastatic disease: the key unmet need in oncology” to MYNERVA project #21267 to MV (MYeloid Neoplasms Research Venture AIRC. A detailed description of the MYNERVA project is available at http://www.progettoagimm.it). This study was supported by Istituto Superiore di Sanità (Rome, Italy), PRIN to MTV and by AIRC 5x1000 call “Metastatic disease: the key unmet need in oncology” to MYNERVA project #21267 to MTV (MYeloid Neoplasms Research Venture AIRC. A detailed description of the MYNERVA project is available at http://www.progettoagimm.it).

## Conflict of Interest

The authors declare that the research was conducted in the absence of any commercial or financial relationships that could be construed as a potential conflict of interest.
