# Supplementary material for: Targeting Lactate Metabolism by Inhibiting MCT1 or MCT4 Impairs Leukemic Cell Proliferation, Induces Two Different Related Death-Pathways and Increases Chemotherapeutic Sensitivity of Acute Myeloid Leukemia Cells
Source: Front Oncol. 2021 Feb 5;10:621458. doi: 10.3389/fonc.2020.621458 (PMC7892602; doi:10.3389/fonc.2020.621458)
Supplement: Supplementary file 1 [file DataSheet_1.pdf]

Supplementary Figure

Figure 1

A

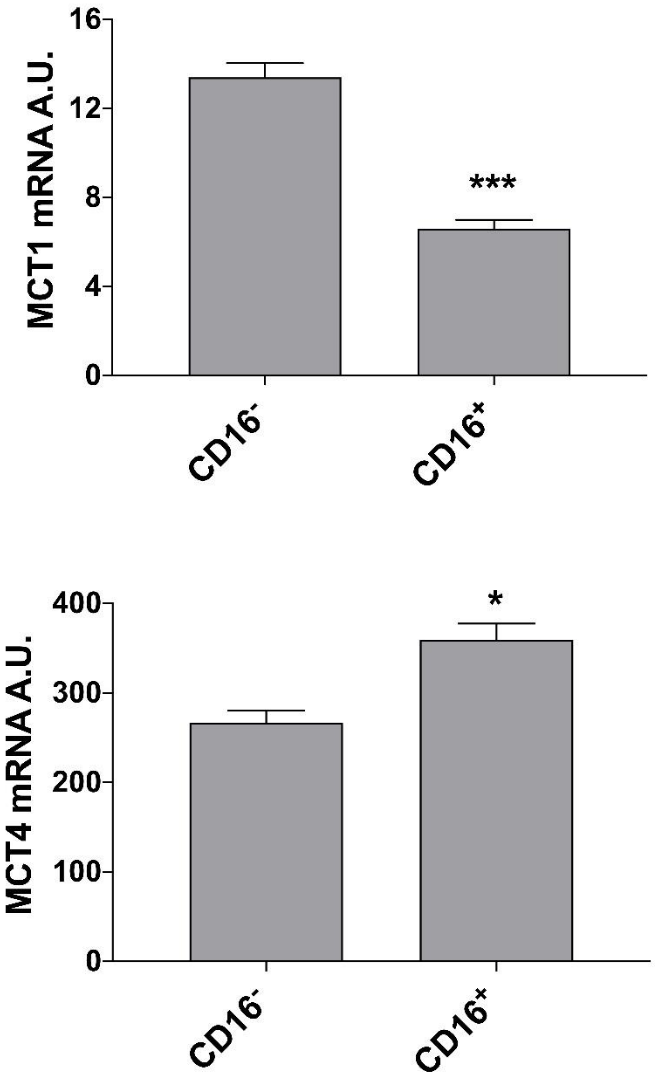

Figure 1. MCT4 high expression in CD16+ monocytic cells. **(A)** qRT-PCR analysis of MCT1 and MCT4 mRNA expression level in the CD16+ subcellular fraction prepared from day 13 Mo differentiating HPCs, as compared to CD16- cells. The results of three independent experiments (mean ± SEM values) are shown; significance * and *** are $p<0.05$ and $p<0.001$ respectively; AU is for arbitrary units.

Figure 2

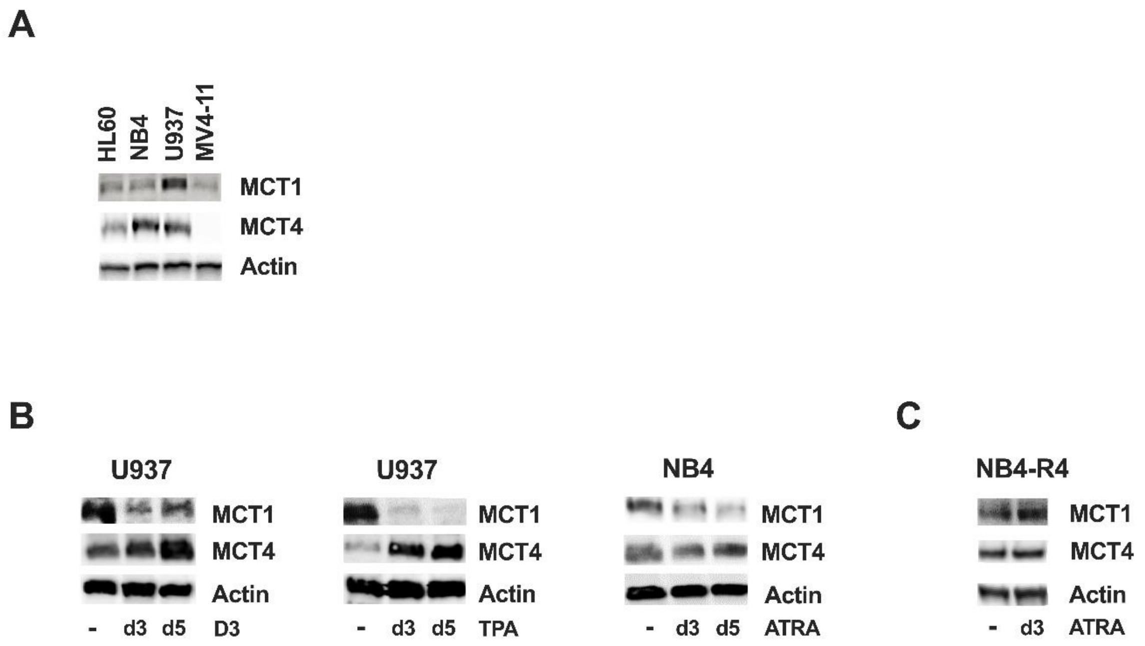

Figure 2. Overexpressed in AML cell lines, MCT1 protein expression decreases while MCT4 protein level increases during monocytic and granulocytic differentiation of leukemic cells. **(A)** Western blot analysis of MCT1 and MCT4 protein expression level in several AML cell lines. **(B)** Western blot analysis of MCT1 and MCT4 protein expression during vitamin D3-induced monocytic differentiation of U937 cells, TPA-induced granulocytic differentiation of U937 cells and ATRA-induced granulocytic differentiation of NB4 cells. **(C)** Western blot analysis of MCT1 and MCT4 protein expression in NB4-R4 cells, resistant to ATRA treatment. **(A-C)** Actin is shown as an internal control. One representative western blot experiment out of three is shown.

Figure 3

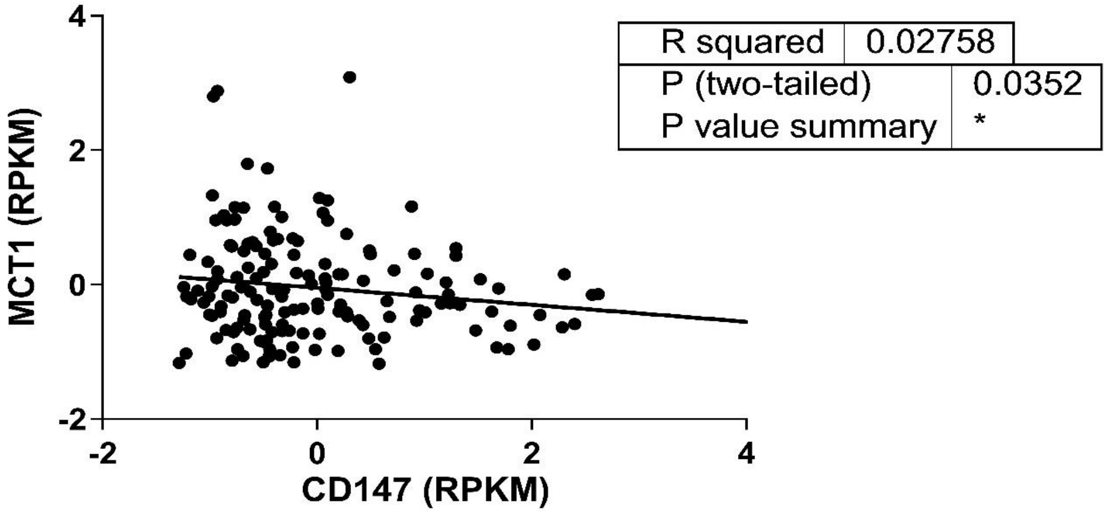

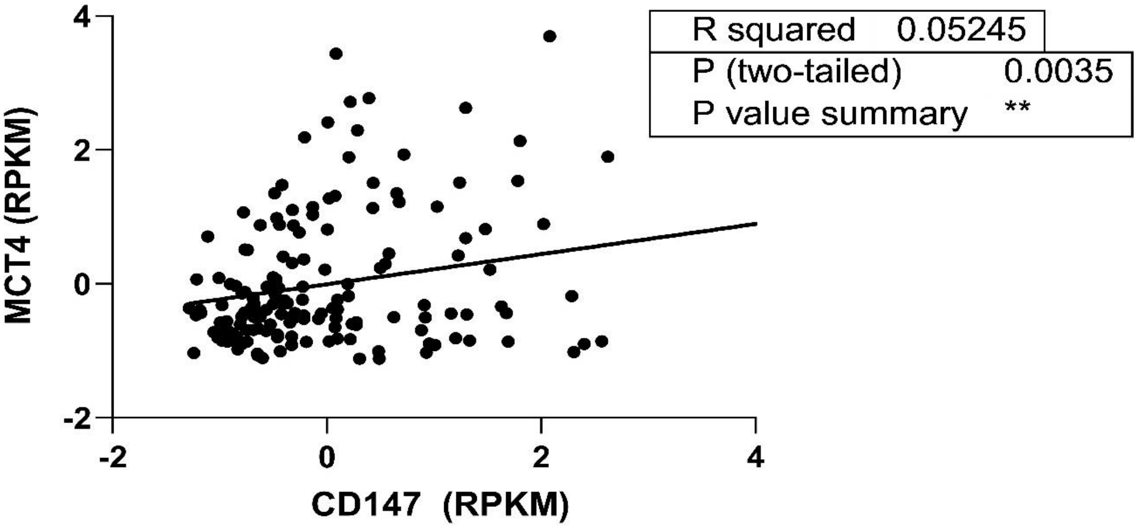

Figure 3. Correlation analysis of MCT1 and MCT4 mRNA expression versus CD147 mRNA expression in AML patients. (***Upper panel***) An inverse correlation is detected between MCT1 and CD147 mRNA expression levels in AMLs, according data from AML samples generated by TCGA Network. ***(Lower panel)*** A positive correlation is detected between MCT4 and CD147 mRNA expression levels in AMLs, according data from AML samples generated by TCGA Network. RPKM is for Reads Per Kilobase of exon per Million mapped reads.

Figure 4

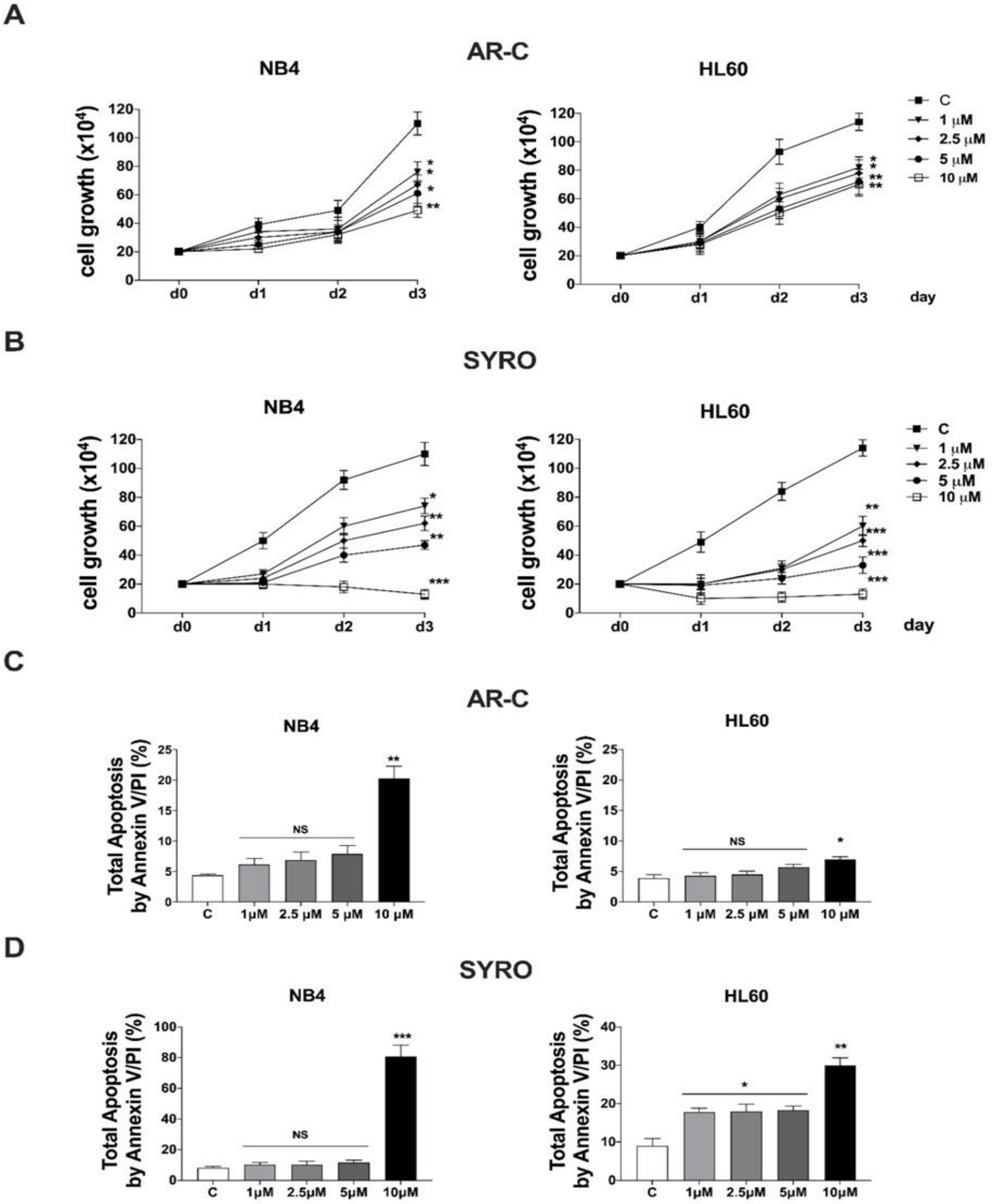

Figure 4. Effects of AR-C and SYRO on cell growth and apoptosis on AML cell lines. **(A)** Dose response analysis of AR-C treatment on NB4 and HL-60 leukemic cell growth, as compared to control cells (c). **(B)** Dose response analysis of SYRO treatment on NB4 and HL-60 leukemic cell growth, as compared to control cells (c). **(C)** Analysis of the effects of AR-C treatment performed for 3 days

on NB4 and HL-60 leukemic cell apoptosis, as compared to control leukemic cell (C). **(D)** Analysis of the effects of SYRO treatment performed for 3 days on NB4 and HL-60 leukemic cell apoptosis, as compared to control leukemic cell (C). **(C, D)** Total apoptosis by annexin V/PI (%) detected by using flow cytometric apoptotic assays, is indicated. **(A-D)** The results of three independent experiments (mean ± SEM values) are shown; significance is *p<0.05; **p<0.01; ***p<0.001; ns is for not significant.
